# Prevalence of Pneumoconiosis in Hubei, China from 2008 to 2013

**DOI:** 10.3390/ijerph110908612

**Published:** 2014-08-25

**Authors:** Ying Xia, Jiafa Liu, Tingming Shi, Hao Xiang, Yongyi Bi

**Affiliations:** 1 School of Public Health, Wuhan University, Wuhan 430079, China; E-Mail: angel.9@sohu.com; 2 Hubei Center for Disease Control and Prevention, 6 Zhuodaoquan North Road, Wuhan 430071, China; E-Mails: L_jiafa@163.com (J.L.); tmingshi@163.com (T.S.); 3 Wuhan University Global Health Institute, Wuhan 430079, Hubei, China

**Keywords:** occupational disease, pneumoconiosis, coal worker's pneumoconiosis (CWP), occupational hazards

## Abstract

We have investigated newly reported pneumoconiosis cases in the province of Hubei, China from 2008 to 2013, to identify the major problems and challenges, and explore possible solutions for its prevention and control. We analyzed the data on new cases of pneumoconiosis from annual reports, including case distributions, patient ages, exposure duration, disease stages, and enterprise types. A total of 3665 new pneumoconiosis cases were reported between 2008 and 2013 in Hubei Province. Coal workers’ pneumoconiosis and silicosis, which accounted for 97.19% of the total, were the most common types. The duration of exposure of 33.32% cases was less than 10 years. Most of the new pneumoconiosis cases worked in industries that produced coal, nonferrous metal, or building materials. About 42.46% of pneumoconiosis cases were from small and medium-sized enterprises. The proportion of cases with combined pneumoconiosis and tuberculosis was 6.6%, and the incidence of tuberculosis was highest in workers with silicosis. The current situation of pneumoconiosis in China is serious. Lack of attention to occupational health, inefficient surveillance, and weak occupational health services may have contributed to the increased new pneumoconiosis cases.

## 1. Introduction

Pneumoconiosis is a progressive and irreversible lung disease caused by occupational inhalation of dusts. Although pneumoconiosis is preventable by environmental dust control, from 1998 to 2002 and from 2003 to 2009 its prevalence has been increasing among workers exposed to occupational dust in China [1]. There were 24,206 new cases of pneumoconiosis diagnosed in 2012, which accounted for 88.28% of all reported occupational diseases. The direct economic losses amount to 8 billion CNY each year [2], and the indirect economic losses amount to 20 billion RMB each year in China [3]. Pneumoconiosis has long been the most prevalent category of diagnosed occupational disease in China [4,5]. There is no specific treatment for pneumoconiosis. Therapeutic whole lung lavage has been used in patients with acute disease, but the prognosis is poor [6].

Hubei Province is pivotal to the strategy for the development of central China, and plays an important role in the transport and industry. The economy has maintained an exceptionally rapid growth rate in recent decades. There are millions of workers behind this economic success and their occupational health is extremely important to Hubei Province. 

Pneumoconiosis is the most prevalent category of diagnosed occupational disease in Hubei Province, it is not only a public health issue, but also a social and economic issue [7]. In addition, workers exposed to high concentrations of occupational dust are particularly at risk of pneumoconiosis, and its pathological changes may continue even after exposure has stopped [8]. Here, we reviewed and analyzed the newly reported pneumoconiosis cases in Hubei Province from 2008 to 2013, and attempted to get more attention from the public health administration.

## 2. Materials and Methods

We extracted annual reports of pneumoconiosis from 2008 to 2013 for Hubei Province from the National Occupational Disease and Occupational Health Information Monitoring System. Through the system, occupational disease notifications are submitted by occupational disease diagnostic physicians through centers for disease control and prevention at the city, county, provincial and national levels to the Ministry of Health. The Ministry of Health then publishes annual statistics of occupational diseases.

In China, the diagnoses for pneumoconiosis were defined by at least three radiologists using the “Diagnostic Criteria of Pneumoconiosis”, which classified pneumoconiosis as suspected or at stage I, II, or III according to the size, profusion and distribution of opacities on chest X-ray [9], and radiography remains an essential tool in the identification of pneumoconiosis [10]. The diagnoses of pneumoconiosis and a comparison between the standard Chinese classification system and International Labour Organisation (ILO) criteria were described by Hodous *et al*. [11]. The Chinese classification system and the accompanying standard radiographic films, were largely analogous to the ILO classification system [12]. 

All pulmonary tuberculosis patients are diagnosed in accordance with the “Diagnostic Criteria for Pulmonary Tuberculosis”. Diagnosis of pulmonary tuberculosis is mainly through laboratory bacteriology examination, combined with chest X-ray film, epidemiological and clinical manifestations. All of the cases were tested by sputum smear.

We analyzed the distribution of pneumoconiosis by type and size of industry, and age and duration of exposure of employees. Curve fitting (d = 0.05) was used to test the linear trend for duration of dust exposure, and median age of employees. Analysis of variance was used to compare age at diagnosis and duration of exposure for difference job titles. All analyses were performed using SPSS software (version 17.0, IBM Corporation, Armonk, NY, USA).

## 3. Results 

In China, pneumoconiosis is classified into 13 categories, including silicosis, coal workers’ pneumoconiosis (CWP) and graphite pneumoconiosis, *etc.* From 2008 to 2013, 3665 cases of pneumoconiosis were reported in Hubei Province, which accounted for 85.61% of all reported occupational diseases. There were 2507, 663 and 495 cases of Stage I, II and III pneumoconiosis, respectively. CWP (2,504 cases, 68.32%) and silicosis (1,058 cases, 28.87%) accounted for most of the new cases of pneumoconiosis. 

The number of cases showed an overall increasing trend (Table 1). There were 32.72% more cases in 2013 (n = 1010) than in 2012 (n = 761). CWP and silicosis were the most common forms of pneumoconiosis and accounted for 97.19% of the cases, indicating that coal and silica dust are currently the most dangerous occupational hazards in Hubei Province. The number of male cases of pneumoconiosis was 73.8 times more than the number of female cases. A total of 15 workers died from pneumoconiosis, and the cumulative mortality rate was 0.41%.

**Table 1:** Age at diagnosis and duration of exposure from 2008 to 2013.

Years	Cases	Age at Diagnosis (Mean ± SD)	Duration of Exposure (Mean ± SD)
2008	387	53.69 ± 10.95	19.74 ± 10.90
2009	209	50.11 ± 9.89	13.21 ± 8.61
2010	782	58.69 ± 10.37	19.94 ± 11.65
2011	516	52.89 ± 11.68	13.36 ± 9.25
2012	761	58.69 ± 11.72	19.26 ± 12.36
2013	1010	54.98 ± 10.55	14.85 ± 8.44
Total	3665	55.83 ± 11.25	17.07 ± 10.80

The average age at diagnosis of individuals with pneumoconiosis in Hubei Province was 56 years, and the average duration of exposure was 17 years. Age and duration of exposure did not show a clear upward or downward time trend, and there were no significant differences over the 6 years studied (Table 2). The maximum age at diagnosis of 73 years was found in individuals with asbestosis pneumoconiosis, while the minimum age at diagnosis of 35 years was found in those with other forms of pneumoconiosis. Individuals aged 45–54 and ≥65 years accounted for 51.09% of the cases of pneumoconiosis. Most of the individuals with stage II or III pneumoconiosis were aged 45–54 years. 

**Table 2:** Incidence of pneumoconiosis according to year of first dust exposure.

First Year of Dust Exposure	Total pneumoconiosis ^*^			CWP ^#^			Silicosis ^+^		
	Cases (%)	Age at diagnosis (mean ± SD)	Length of service (mean ± SD)	Cases (%)	Age at diagnosis (mean ± SD)	Length of service (mean ± SD)	Cases (%)	Age at diagnosis (mean ± SD)	Length of service (mean ± SD)
<1960	261 (7.21)	75.40 ± 4.27	25.37 ± 12.03	121 (4.83)	75.11 ± 4.38	29.47 ± 8.62	130 (12.29)	75.74 ± 3.88	21.33 ± 13.72
1960–	505 (13.78)	67.07 ± 4.81	23.15 ± 11.26	329 (13.14)	67.21 ± 4.69	27.02 ± 8.81	168 (15.88)	66.63 ± 4.91	15.39 ± 11.66
1970–	1017 (27.75)	61.36 ± 5.12	23.04 ± 9.30	788 (31.47)	61.38 ± 4.96	23.58 ± 8.65	211 (19.94)	61.13 ± 5.25	21.11 ± 11.22
1980–	516 (14.08)	49.63 ± 6.56	19.05 ± 6.29	367 (14.66)	46.12 ± 5.33	18.75 ± 6.03	128 (12.10)	48.61 ± 6.32	19.25 ± 6.86
1990–	517 (14.11)	46.01 ± 6.92	12.04 ± 4.70	346 (13.82)	45.98 ± 6.60	12.89 ± 4.19	143 (13.52)	46.28 ± 7.66	9.56 ± 5.16
2000–	849 (23.17)	46.27 ± 6.15	5.62 ± 2.78	553 (22.08)	46.42 ± 5.75	5.82 ± 2.80	278 (26.28)	46.34 ± 6.68	5.18 ± 2.68
Total	1058 (100)	56.39 ± 12.29	14.26 ± 11.13	2504 (100)	46.25 ± 6.05	18.21 ± 10.49	1058 (100)	56.39 ± 12.29	14.26 ± 11.13

Age at diagnosis: F^*^ = 670.09, p^*^ < 0.05; F^#^ = 1309.50, *p*^#^ < 0.05; F^+^ = 70.09, *p* ^+^ < 0.05; Length of service: F^*^ = 113.37, p^*^ < 0.05; F^#^ = 677.34 , *p*^#^ < 0.05; F^+^ = 113.37, *p*^+^ < 0.05.

**Table 3:** Duration of exposure and age at diagnosis by job title.

Job Title	Cases (%)	Age at Diagnosis (Mean ± SD)	Duration of Exposure (Mean ± SD)
Simple coal mining	538 (14.68)	52.75 ± 9.34	16.12 ± 9.39
Coal mining	765 (20.87)	54.57 ± 10.98 ^*^	16.35 ± 9.69
Coal mixture	303 (8.27)	54.78 ± 10.90 ^*^	19.33 ± 11.62 ^*^
Only tunneling	339 (9.25)	57.96 ± 9.95 ^*^	18.81 ± 10.70 ^*^
Mainly tunneling	659 (17.98)	58.07 ± 10.98 ^*^	19.49 ± 11.05 ^*^

Compared to only coal mining, ^*^ *p* < 0.05.

**Table 4:** Cases of pneumoconiosis complicated with pulmonary tuberculosis.

Pneumoconiosis categories	Reported cases (TB cases)	Incidence (%)	Stage I cases (TB cases)	Incidence (%)	Stage II cases (TB cases)	Incidence (%)	Stage III cases (TB cases)	Incidence (%)
CWP	2504 (165)	6.59	1730 (141)	8.15	449 (17)	3.79	325 (7)	2.15
Silicosis	1058 (76)	7.18	683 (55)	8.05	209 (15)	7.18	166 (6)	3.61
Other categories	103 (1)	3.23	94 (1)	3.45	5 (0)	0.00	4 (0)	0.00
Total	3665 (242)	6.60	2507 (197)	7.86	663 (32)	4.83	495 (13)	2.63

Individuals with duration of exposure <10 years accounted for 33.32% of all cases of pneumoconiosis; 10–19 years accounted for 27.86%; 20–29 years accounted for 21.23%; and ≥30 years accounted for 17.60%. The minimum median duration of exposure of 4 years was in the group with cement pneumoconiosis, while the maximum median duration of exposure of 24 years was among those with welder pneumoconiosis. The duration of exposure in individuals with silicosis showed a slight increasing trend from 2011 to 2013. However, there was no obvious trend in those with CWP or for pneumoconiosis overall.

Stage I pneumoconiosis cases accounted for 68.40% of the total. The age at diagnosis and duration of exposure were higher in individuals with Stage I pneumoconiosis compared with stage II and III disease. The age at diagnosis and duration of exposure were higher for Stage III than stage II pneumoconiosis. Significant differences were found between the three stages (*p* < 0.05).

Table 2 shows the newly reported cases of pneumoconiosis according to the first year of dust exposure. The earliest year of dust exposure was 1941, whereas the latest year was 2012. All pneumoconiosis cases were divided into six categories according to the period of dust exposure (before 1960, 1960–1969, 1970–1979, 1980–1989, 1990–1999, after 2000). Most cases were seen among individuals who were first exposed to dust in 1970–1979 and after 2000. The average age and the duration of exposure of dust exposure decreased, significant differences were found for each group.

In Hubei Province, pneumoconiosis occurred most frequently in the coal mining (35.55%), tunneling (27.23%) and coal mixture (8.27%) industries. The age at diagnosis and duration of exposure of simple coal miners were significantly shorter than those of other jobs (*p* < 0.05) (Table 3).

All 3,665 cases of pneumoconiosis were distributed among 23 industries, with 74.10% reported in the coal industry. Others were mainly from the nonferrous metals (198 cases, 5.40%), building materials (190 cases, 5.18%) and geology and mineral resources (151 cases, 4.12%) sectors. About 42.46% of pneumoconiosis cases were from small and medium-sized enterprises (SMEs), whereas 36.45% were from large enterprises, and 20.93% cases from enterprises of unknown size. Stage II and III pneumoconiosis accounted for 18.09% and 13.51% of all the cases, respectively.

The proportion of individuals with pneumoconiosis complicated with pulmonary tuberculosis was 6.60% (Table 4). The proportion decreased with increasing stage of pneumoconiosis. Among the 13 forms of pneumoconiosis, complication with pulmonary tuberculosis was most common with silicosis (7.18%).

## 4. Discussion and Conclusions

High-quality data on occupational diseases are also essential for employers, occupational safety and health (OSH) regulators, social security institutions, OSH professionals and other stakeholders to fulfill their obligations for the prevention and control of work-related diseases. They are also important in setting priorities and targets for safety and health at work, in designing relevant training and education programs, in improving rehabilitation, and for the compensation process in cases of occupational disease.

Only a few countries (China, USA, United Kingdom, New Zealand, Germany, Finland, *etc.*) have comprehensive systems for collecting occupational disease data [13].The data reported is different, and some systems are reported by employers, some systems are reported by doctors, and some by the workers or their household. In China, the reported data is provided mainly by occupational health physicians. The main objective of the system is to provide comprehensive, reliable data on occupational diseases, and occupational health physicians are responsible for reporting related data. Based on the information from the system, the government could devise strategies and programs to protect workers’ health, prevent occupational diseases, enhance workers’ work ability and productivity, and compensate the victims of occupational disease.

The system comprises general rules, uniform procedures, institutional arrangements, obligations, responsibilities, rights and duties for the relevant parties, such as the national authority, occupational health services hospitals, and employers. The Notification Card for Occupational Diseases in China comprises ID number of case, employer information, type of notification, name of worker, date of birth, sex, exposure duration, date of diagnosis, date of death, cause of death, notification institution, manager of institution, date of notification, *etc.*

The National Health and Family Planning Commission of China announces the occupational diseases cases annually, but its content is very limited. We hope the government will provided more information data to the public in the future.

The occupational exposure limit for silica dust in China was established using the total dust concentration, based on the silica proportion in the total dust. The permissible time-weighted average concentration of total dust in China is 1, 0.7, 0.5 mg/m^3^ for dust that contains ≤50%, ≤80%, >80% of silica, respectively. It is not required to collect the data of dust exposure for each individual in China, in fact this data is very difficult to achieve because many enterprises do not monitor dust concentrations, and many migrant workers work in SMEs and perform hazardous jobs without sufficient protective equipment.

Hubei Province plays an important role in the economic rise of central China. The rapid economic growth in recent decades was accompanied by an increase in the number of cases of pneumoconiosis. Our study indicated that the current situation with pneumoconiosis in Hubei was serious, with the number of cases showing a significantly increasing trend. A similar trend has also been observed in Hunan [14], Jiangsu [15] and Zhejiang [16] provinces in the past years. Possible reasons include the absence of effective prevention and control measures, and ineffective surveillance systems.

Even if effective methods are implemented to decrease occupational dust in the workplace, this trend is unlikely to be arrested or reversed within short time, because of the long incubation period of pneumoconiosis. Workers who have been exposed to occupational dust, especially low concentrations, may develop symptoms of pneumoconiosis 15 to 20 years later. This may make the pneumoconiosis situation even worse. 

There was a survey to investigate the number of workers exposed to dust in Hubei Province in 2012, however, due to the survey design defects and inaccuracy of the information obtained from migrant workers, we do not think the number of exposed to dust workers (denominator) from that survey can be adopted or used as a reference. In fact, it is very hard to check the accurate number of these workers for these kinds of reasons

CWP is a chronic occupational lung disease that is caused by long-term inhalation of coal dust. Exposure can trigger inflammation of the alveoli, cause irreversible lung damage, and subsequently CWP. Thus, CWP is a preventable occupational lung disease but difficult to cure. In China, CWP accounted for 44.2% of the total number of pneumoconiosis cases from 1997 to 2009, and 51.25%–53.01% from 2010 to 2012. Based on our results, CWP accounted for 68.32% of all pneumoconiosis cases from 2008 to 2013 in Hubei, the ratio is significantly higher than the national, Zhejiang (7.6%), Hunan (40.40%) and Jiangsu (22.99%) and other provinces’ averages, indicating that the situation is even more serious in Hubei Province. 

Several factors increase workers’ risk of developing pneumoconiosis, such as the concentration of respirable coal dust, coal dust particle size and composition, duration of exposure, age, and work environment and practices [17,18]. In addition, the increase in CWP has resulted from increased work hours and workloads [19]. Without effective protection for workers, an increasing number of them are at risk for CWP because there is increased demand for coal [20].

We found that the coal industry accounted for 74.10% of cases, and the pooled prevalence from high to low for the different types of work was coal mining, tunneling and coal mixture work, respectively. Among coal miners, age at diagnosis of pneumoconiosis was lowest and duration of exposure was shortest, implying that they were the most seriously affected by occupational dust hazards. Therefore, we should focus on strengthening management of the coal industry to decrease morbidity. Coal mining companies should improve the monitoring and reduction of dust in tunneling and mining areas. 

In Hubei Province, most pneumoconiosis cases were among workers who were first exposed to dust in 1970 to 1979 and after 2000. The average age at diagnosis and the duration of exposure of dust exposure decreased which implies that the risk of dust become more and more serious.

A total of 42.46% of pneumoconiosis cases were from SMEs in Hubei. Most enterprises frequently fail to carry out their legally binding responsibilities to manage occupational hazards in the workplace. In particular, SMEs generally give insufficient attention to pneumoconiosis prevention and dust control. According national law issued in 1963, all dust-exposed workers should have opportunity to receive physical examination every 1 to 2 years, and employers should bear the cost [21]. However, because of inadequate supervision, many employers did not provide enough opportunities, many workers were diagnosed stage II and III pneumoconiosis when first diagnosed, and the number of cases of stage II and III pneumoconiosis was high, which should be dealt with efficiently to prevent further complications.

The occupation disease prevention law decrees that the fees of diagnosis, treatment and recovery of occupational disease patients should be paid by the worker injury insurance company. If the employer does not participate in work injury insurance, the cost is paid by the employer. If the enterprises have closed down or declared bankruptcy or the patients are migrant workers who haven’t make contracts with enterprises, and are unable to confirm the labor relationship, occupational disease patients can request medical and life assistance from the civil affairs departments of the local governments.

The proportion of cases of pneumoconiosis complicated with pulmonary tuberculosis was 6.60% in Hubei Province, which decreased by 9.22% compared with the rate of 15.82% in 1986 (national epidemiological survey results). However, it was higher than the prevalence of active tuberculosis from the Fourth National Chinese Tuberculosis survey in 2000 (0.37%) [22]. The proportion of cases of pneumoconiosis stage I, II and III complicated with pulmonary tuberculosis was 6.96%, 9.91% and 31.97%, respectively, which was lower than 14.82%, 14.74% and 34.60% in 1986. The main reasons for this reduction are: (1) the morbidity of pulmonary tuberculosis shows a slowly decreasing tendency and the efficiency of clinical treatment has been improved, and (2) the increasing physical quality of the nation, together with the improvement of the sanitary conditions and medical care led to a lower incidence of pneumoconiosis complicated with pulmonary tuberculosis. The amount of compensation is not the same between pneumoconiosis complicated with pulmonary tuberculosis patients with pneumoconiosis patients, as the compensation for the former is higher than for the latter. 

The China Occupational Disease Prevention Plan (2009 to 2015) clearly pointed out that “occupational disease prevention and control focus on coal workers' pneumoconiosis, silicosis and asbestosis, integrate measures dust hazard” [23]. Our study should be useful for occupational regulatory agencies to design appropriate policies to reduce this highly preventable disease among dust-exposed workers, including proactive reduction of exposure to dust, more stringent regulation of SMEs, and more stringent monitoring of coal mining activities in Hubei. In addition, faced with the current situation of pneumoconiosis, combined with the epidemiological characteristics of pneumoconiosis, the government of Hubei Province should formulate a pneumoconiosis control strategy, in order to prevent the occurrence of pneumoconiosis, and protect the health of workers.
